# Position of different nebulizer types for aerosol delivery in an adult model of mechanical ventilation

**DOI:** 10.3389/fmed.2022.950569

**Published:** 2022-10-10

**Authors:** Haijia Hou, Dongyang Xu, Bing Dai, Hongwen Zhao, Wei Wang, Jian Kang, Wei Tan

**Affiliations:** Department of Respiratory and Critical Care Medicine, The First Hospital of China Medical University, Shenyang, China

**Keywords:** aerosol delivery, jet nebulizer, vibrating mesh nebulizer, position, inspiratory synchronized

## Abstract

**Background:**

The optimal positions of different types of nebulizer for aerosol delivery remain unclear.

**Methods:**

Three ICU ventilators employing three types of nebulizer were separately connected to a simulated lung to simulate nebulization during invasive ventilation. Assist/control-pressure control (A/C-PC) mode was utilized, with inspiratory pressure (Pi) set to 12 cmH_2_O and positive end expiratory pressure (PEEP) set to 5 cmH_2_O, and with a target Vt of 500 ml. The bias flow of all the ventilators was set to 2 L/min. The three nebulizers were the continuous jet nebulizer (c-JN), the inspiratory synchronized jet nebulizer (i-JN), and the vibrating mesh nebulizer (VMN). The five nebulizer positions were as follows: at the Y-piece (position 1) and 15 cm from the Y-piece (position 2) between the endotracheal tube and the Y-piece, at the Y-piece (position 3) and 15 cm from the Y-piece (position 4) in the inspiratory limb; and at the humidifier inlet (position 5). Aerosols were collected with a disposable filter placed at the simulated lung outlet (*n* = 3) and were measured by UV spectrophotometry (276 nm). The measurements were compared under different experimental conditions.

**Results:**

The aerosol delivery of c-JN, i-JN, and VMN was 5.33 ± 0.49~11.12 ± 0.36%, 7.73 ± 0.76~13.75 ± 0.46% and 11.13 ± 56–30.2 ± 1.63%, respectively. The higher aerosol delivery: for c-JN~Positions 2 (10.95 ± 0.15%), fori-JN~Positions 1 or 2 (12.91 ± 0.88% or 13.45 ± 0.42%), for VMN~Positions 4(29.03 ± 1.08%); the lower aerosol delivery: for c-JN~Positions 1, 3 or 5, fori-JN~Positions 4 or 5, for VMN~Positions 5.

The highest aerosol delivery:For c-JN at Position 2 (10.95 ± .15%), for i-JN at Position 1 or 2 (12.91 ± .88% or 13.45 ± .42%), for VMN at Positions 4 (29. 03 ± 1.08%); the lower aerosol delivery: for c-JN at Positions 1, 3 or 5, for i-JN at Positions 4 or 5, for VMN at Positions 5. The highest aerosol deliveryof c-JN was lower than that of i-JN while the VMN was the highest (all *P* < .05). However, no differences were observed between the highest aerosol delivery with c-JN and the lowest aerosol delivery with i-JN. Similar results were found between the lowest aerosol delivery with VMN and the highest aerosol delivery with c-JN /i-JN in the Avea ventilator. There were no differences in the highest aerosol delivery of each nebulizer among the different ventilators (all *p* > 0.05).

**Conclusion:**

During adult mechanical ventilation, the type and position of nebulizer influences aerosol delivery efficiency, with no differences between ventilators.

## Introduction

Delivery of therapeutic aerosol is an important component of treatment in many respi ratory disorders (1, 2), and it is commonly administered in mechanically ventilated patients (3–5). Two types of nebulizer, including the jet nebulizer (JN) and the vibrating mesh nebulizer (VMN), are now among the most commonly employed means of delivering liquid formulations of medications to mechanically ventilated patients (2, 4, 6–8). There are two main modes of nebulization used in clinics, namely, continuous and inspiratory synchronized. Inspiratory synchronized JN (ventilator-integrated JN, i-JN) systems have long been known to improve inhaled dosage relative to continuous JN (c-JN) (3, 9–11), but inspiratory synchronized VMNs are currently undergoing investigation (12) and are not commercially available.

Early studies found that aerosol delivery with a nebulizer to the distal airways/alveoli of a mechanically ventilated patient is influenced by ventilator settings for gas flow, residual volume, and bias flow, with the type of nebulizer employed, mode of nebulization, and position of the nebulizer in the circuit having particular importance (4). However, the optimal positions of different nebulizer types for aerosol delivery remain unclear. Ari et al. found that inhaled doses with the nebulizer placed at the inlet of the humidifier were similar to those with the nebulizer placed close to the patient with bias flow (13). For continuous VMN, the optimal position was the inlet of the humidifier with bias flow (13). Since that time, continuous nebulizers have been recommended to be placed at the inlet/outlet of the humidifier or far from the patient with bias flow (4, 14–16). For i-JN, only one study has compared two nebulizer positions and found that the optimal position was before the Y-piece in the inspiratory limb (17). Thus, this position has been recommended to be placed during invasive ventilation (4). Nonetheless, in two survey studies, the preferred nebulizer position was between the endotracheal tube (ETT) and the Y-piece or at the inspiratory limb just before the Y-piece regardless of nebulizer type (7, 8).

However, in all of these bench studies, only two or three positions were compared for each nebulizer type/mode. No consensus has emerged on the most favorable position for drug deposition in a laboratory setting. More importantly, to our knowledge, there are no studies comparing the effects of different ventilators on aerosol delivery. Here, we conducted a comprehensive comparison of aerosol delivery with three nebulizer types and five positions in three ICU ventilators during invasive ventilation *in vitro* to investigate the position of c-JN, i-JN, and VMN, and the effect of different nebulizer types/modes and different ventilators on aerosol delivery.

## Materials and methods

### Bench model setup

The Active Servo Lung 5 000 respiratory simulation system (ASL5000; IngMar, United States) is a precision breathing simulator that contains a piston that moves in a cylinder and can simulate different breathing patterns in candidates for invasive ventilation. The following parameters were applied for the simulated lung (18–20): compliance 60 ml/ cmH_2_O, inspiratory resistance 10 cmH_2_O/L/s, expiratory resistance 15 cmH_2_O/L/s, and maximum inspiratory pressure drop −8 cmH_2_O. To simulate the profile of the negative pressure created by respiratory muscles, 5% of the respiratory cycle time was spent in active inspiration, 3% in end inspiratory hold, and 15% in return of pressure to baseline. The breathing rate was set at 15 breaths/min.

Three ICU ventilators were used: V300 (Drägerwerk AG & Co. KGaA, Lübeck, Germany), Avea (Vyaire Medical, Solon OH 44,139, America), and Servo-s (Maquet Critical Care, Solna, Sweden). Of these, both the V300 and Avea ventilators have i-JN, whereas the Servo-s ventilator does not. The ventilators were connected to a humidifier (MR850, Fisher & Paykel Healthcare) and an adult-sized, dual-limb, heated wire circuit (RT100, Fisher & Paykel Healthcare), and then a Y-piece was connected to the lung model with a 30-cm long, 2.2-cm diameter tube. The humidifier was used with distilled water, set to invasive mode (37°C), and was turned on for a minimum of 20 min before the start of the experiment. The assist/control-pressure control (A/C-PC) mode was utilized, with inspiratory pressure (Pi) set to 12 cmH_2_O and positive end expiratory pressure (PEEP) set to 5 cmH_2_O, and with a target Vt of 500 ml. The bias flow of all the ventilators was set to 2 L/min.

Three nebulization modes were used: (1) c-JN where the nebulizer (1884; Teleflex, Mexico) was driven *via* compressed oxygen and the flow meter (Pacific Medical, Taiwan) maintained the oxygen flow at 8 L/min; (2) i-JN where the nebulizer (1884; Teleflex, Mexico) was driven directly with the V300 and Avea ventilators during the inspiratory phase; and (3) VMN (Aeroneb^®^ Pro; Aerogen, Ireland).

**Figure 1:**
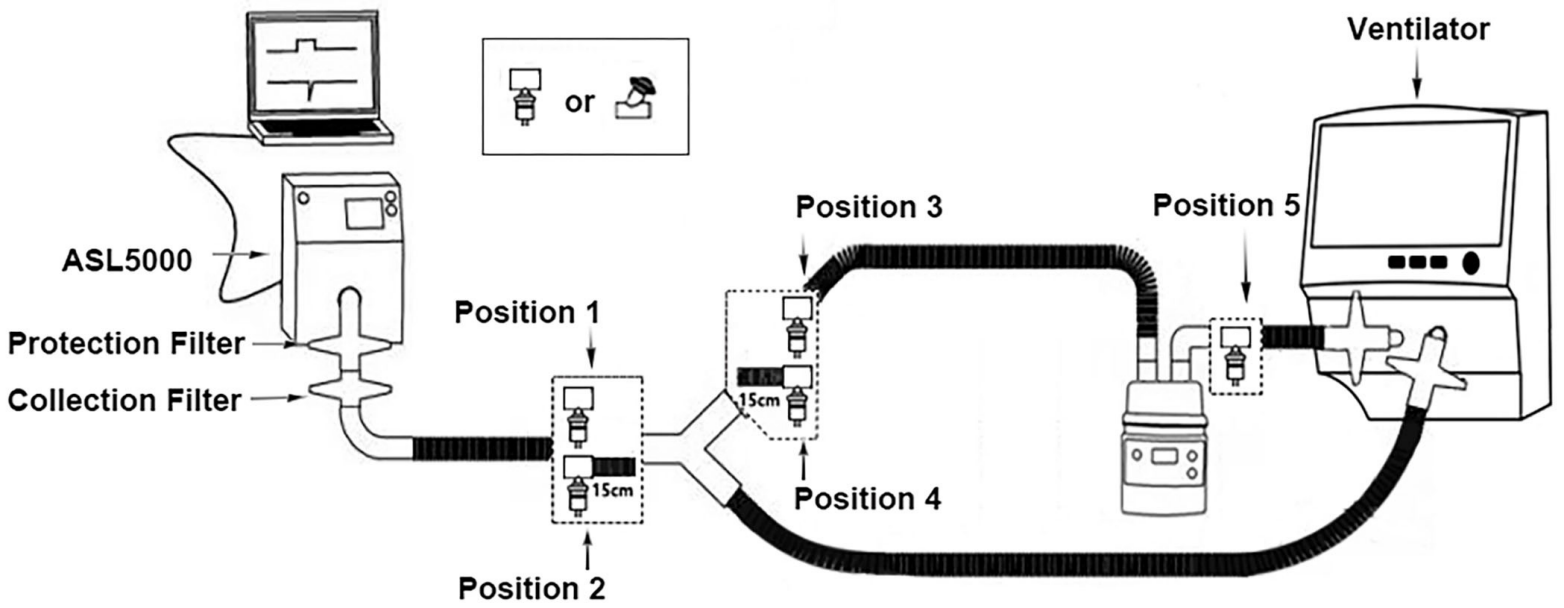
Schematic diagram of the experimental setup. Position 1, at the Y-piece, between the endotracheal tube and the Y-piece; position 2, 15 cm from the Y-piece, between the endotracheal tube and the Y-piece; position 3, at the Y-piece in the inspiratory limb; position 4, 15 cm from the Y-piece in the inspiratory limb; position 5, at the humidifier inlet.

The bench model is illustrated in Figure 1. The ventilators and the nebulizers were connected to the simulated lung through a humidifier and an adult-sized, dual-limb, heated wire circuit to simulate nebulization during invasive ventilation. Five nebulizer positions were adopted as follows: at the Y-piece (position 1), 15 cm from the Y-piece (position 2) between the endotracheal tube (ETT) and the Y-piece, at the Y-piece (position 3), 15 cm from the Y-piece (position 4) in the inspiratory limb, and at the humidifier inlet (position 5).

### Aerosol delivery and measurement

For nebulization, a 1-ml solution of 0.5% salbutamol (Ventolin; GlaxoSmithKline, Australia) was diluted with 3 ml normal saline, and the solution was placed into the nebulizer. Aerosol particles were intercepted with a collection filter (REF19212; Teleflex Medical, Malaysia) placed at the simulated lung outlet. A stopwatch was used to record the nebulization time; the nebulization of JN was considered successfully completed when no visible evidence of nebulization was seen for 30 s; the nebulization of VMN was considered successfully completed when there was no visible evidence of nebulization. After nebulization, the ventilators and the simulated lung were turned off for at least 1 min. The experiments were run thrice under each experimental condition.

Following each nebulization, the filter was washed with 10 ml normal saline to collect the aerosol. The filter was shaken using a vortex shaker (XW-80A; Huxi, Shanghai) for 1 min to fully mix the normal saline and salbutamol aerosol particles. The washing solution was placed in a 1-ml quartz glass cup, and a UV spectrophotometer was used to measure the absorbance of the solution at a wavelength of 276 nm. The absorbance at this wavelength had a linear relationship with the concentration of the salbutamol solution over the range of 0 to 0.1 mg/ml, and the slope of the standard curve was 0.1426 (R^2^ = 0.99). The standard curve was used to calculate the corresponding salbutamol concentration and amount. All the experiments were performed by the same investigator (XD).

### Statistical analysis

All the data were analyzed using GraphPad PRISM (GraphPad Software, San Diego, CA). Aerosol delivery and ventilator performance are presented as means and standard deviations. A one-way analysis of variance (ANOVA) was conducted to compare the effects of the different interfaces on aerosol delivery and ventilator performance. The LSD-t method was used for pairwise comparison. Two-tailed *p-*values < 0.05 and changes > 10% indicated statistical and clinical significance for comparisons of aerosol delivery and ventilator performance (18–20).

## Results

The aerosol delivery of c-JN, i-JN, and VMN was 5.33 ± 0.49~11.12 ± 0.36%, 7.73 ± 0.76~13.75 ± 0.46% and 11.13 ± 56~30.2 ± 1.63%, respectively. The mean aerosol delivery times were 10.68 ± 0.64, 46.43 ± 10.33, and 13.47 ± 0.67 min, respectively.

### Performance of c-JN

**Figure 2:**
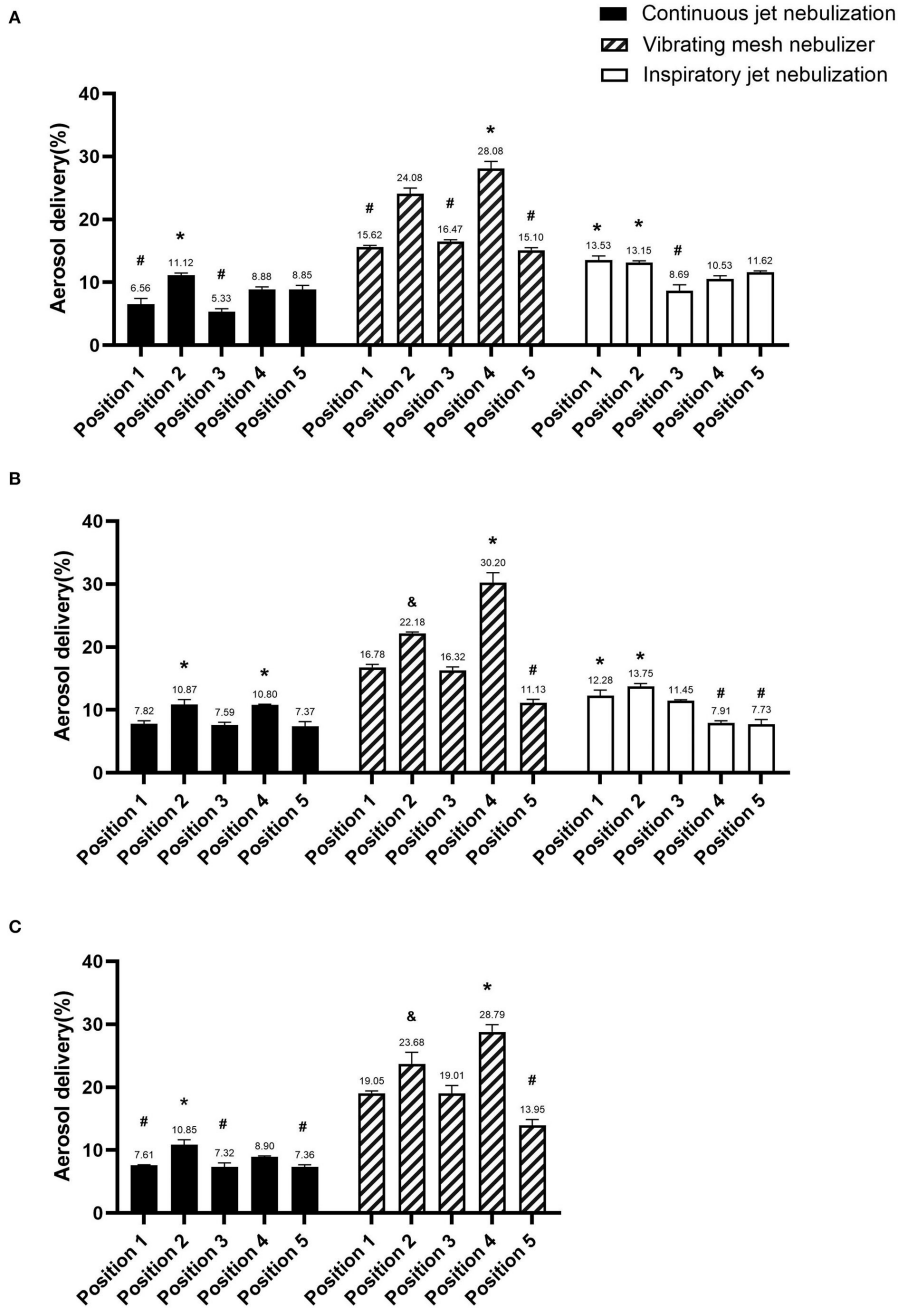
Comparison of aerosol delivery of the different nebulizer types in the three ventilators. **(A)** V300 ventilator, **(B)** Avea ventilator, and **(C)** Servo-s ventilator. *Significantly higher than other positions (*p* < 0.05), ^&^significant differences with other positions (*p* < 0.05), and ^#^significantly lower than other positions (*p* < 0.05).

Aerosol delivery was higher when the nebulizer was placed in position 2 (10.95 ± 0.15%) than in the other positions (all *p* < 0.001), but there were no differences in aerosol delivery among the different ventilators (V300 11.12 ± 0.36%, Avea 10.87 ± 0.79%, and Servo-s 10.85 ± 0.79%) (all *p* > 0.05) (Figure 2). Aerosol delivery was lowest when the nebulizer was placed in positions 1 (7.33 ± 0.67%), 3 (6.75 ± 1.24%), and 5 (7.86 ± 0.86%) (all *p* < 0.05).

### Performance of I-JN

Aerosol delivery was higher when the nebulizer was placed in position 1 (12.91 ± 0.88%) or 2 (13.45 ± 0.42%) than in the other positions (all *p* < 0.001), but there were no differences in aerosol delivery among the different ventilators (V300 13.53 ± 0.68, 13.15 ± 0.25%; Avea 12.28 ± 0.87, 13.75 ± 0.46%; respectively) (all *p* > 0.05) (Figure 2). Aerosol delivery was lowest when the nebulizers were placed in positions 4 (9.22 ± 1.85%) and 5 (9.68 ± 2.76%) (all *p* < 0.001).

### Performance OfVMN

Aerosol delivery was higher when the nebulizer was placed in position 4 (29.03 ± 1.08%) than in the other positions (all *p* < 0.001), but there were no differences in aerosol delivery among the different ventilators (V300 28.08 ± 1.12%, Avea 30.2 ± 1.63%, and Servo-s 28.79 ± 1.16; respectively) (all *p* > 0.05) (Figure 2). Aerosol delivery was lowest when the nebulizer was placed in position 5 (13.39 ± 2.04%) (all *p* < 0.001).

### Comparison of aerosol delivery among the three nebulizer types

The mean aerosol delivery of c-JN, i-JN, and VMN were 8.48 ± 1.76, 11.07 ± 2.27, and 20.03 ± 5.85%, respectively, with no significant difference between c-JN and i-JN, but both of them had significantly lower values than VMN.

The highest aerosol delivery of c-JN (position 2, 10.95 ± 0.15%) was lower than that of i-JN (position 1, 12.91 ± 0.88%; position 2, 13.45 ± .42%), while VMN had the highest (position 4, 29.03 ± 1.08%) (all *p* < 0.05). However, no differences in aerosol delivery were observed between the highest aerosol delivery with c-JN (position 2, 10.95 ± 0.15%) and the lowest aerosol delivery with i-JN (position 4, 9.22 ± 1.85%, *p* = 0.5; position 5, 9.68 ± 2.76%, *p* = 0.74). Similar results were found between the lowest aerosol delivery with VMN (position 5, 11.13 ± 0.56%) and the highest aerosol delivery with c-JN (position 2, 10.87 ± 0.79%, *p* = 0.66)/i-JN (position 1, 12.28 ± 0.87%, *p* = 0.13) in the Avea ventilator.

## Discussion

In this study, five nebulizer positions of three nebulizer types and three ICU ventilators were chosen to compare aerosol delivery during invasive ventilation, and significant differences were observed among the different positions. We found that it is very important to place the nebulizer in the appropriate position in the circuit, i.e., the aerosol delivery of VMN is not always higher than that of c-JN/i-JN in some nebulization positions. The highest aerosol delivery of c-JN, i-JN, and VMN was observed when the nebulizers were placed in positions 2 (15 cm before the Y-piece), 1 (close to the Y-piece) or 2 (15 cm before the Y-piece), and 4 (15 cm from the Y-piece in the inspiratory limb), respectively. Besides, no differences were seen in the highest aerosol delivery for the nebulizer types among the different ventilators.

Similar to a previous study (21), we found the highest aerosol delivery of JN to be lower than that of i-JN, and that the delivery of VMN was the highest. VMN has gained clinical popularity because of its quiet operation, relatively high efficiency, low residual volume, minimal influence on ventilation parameters, minimal disruption of ventilation, and no release with a closed-circuit VMN compared to JN (22–25). However, many factors influence aerosol delivery during invasive mechanical ventilation, including the position of the nebulizer in the ventilator circuit (4). Our study found no differences in delivery between the lowest aerosol delivery with VMN and the highest aerosol delivery with c-JN/i-JN. Thus, it is important to place the nebulizer in the proper position to ensure the highest aerosol delivery for a given ventilator and nebulizer type.

Two studies (13, 26) have compared aerosol delivery with different nebulizer positions of c-JN during invasive ventilation (Table 1). In one study with bias flow, no better position of aerosol delivery was found (13), and in another study without bias flow, the better nebulizer position was 15 cm from the ventilator (26). This was because it was farther from the endotracheal tube and therefore improved aerosol delivery, since the ventilator tube acts as a spacer in which the aerosol accumulates between breaths. It is recommended that continuous nebulizers be placed at the inlet/outlet of the humidifier or far away from the patient with bias flow (4, 14–16). However, we found that the better nebulizer position was position 2 (15 cm before the Y-piece), which is a relatively new position and easy to utilize in the clinical context. The 15-cm tube may also act as an aerosol spacer and reduce aerosol from being expelled from the expiratory limb directly during the expiratory phase.

**Table 1:** Studies about the position of different nebulizer types during invasive ventilation *in vitro* study.

	**Author, year**	**Ventilator setting**	**Heated-humidity (°C)**	**Bias flow (L/min)**	**Aerosal delivery of different nebulizer position (%)**							**Optimal nebulizer position**
					**Between the ETT and the Y-piece (position 1)**	**From the Y piece in the inspiratory limb**			**Humidifie**		**15cm from ventilator (position 5)**	
						**Before Y-piece (position 3)**	**15 cm (position 4)**	**30/45 cm**	**Outlet**	**Inlet**		
c-JN	Ari, 2010 (26)	Vt 500, PEEP 5, *f* 15	35 ± 1°C	0	4.7 ± 0.5		3.6 ± 0.2				6.0 ± 0.1	15 cm from ventilator
			*N*		7.6 ± 0.9		9.7 ± 1.5				14.7 ± 1.5	15 cm from ventilator
	Ari, 2010 (13)	Vt 500, PEEP 5, *f* 20	35 ± 1°C	2			4.7 ± 0.1			5.2 ± 0.2		
				5			4.0 ± 0.1			4.7 ± 0.4		
			*N*					10.4 ± 0.8				
i-JN	O'Doherty, 1992 (17)	MV 6-15, *f* 10-20, Ti 20-50%	Bennet Cascade II	MD	5.4 ± 0.15	8.0 ± 0.94						Before the Y piece in the inspiratory limb
VMN	Ari, 2010 (26)	Vt 500, PEEP 5, *f* 15, Ramp flow pattern, peak flow 60	35 ± 1°C	0	12.8 ± 0.5		16.8 ± 2.6				8.4 ± 2.1	15 cm from the Y-piece in the inspiratory limb
			*N*		14.5 ± 1.0		30.2 ± 1.0				24.2 ± 1.2	
	Ari, 2010 (13)	Vt 500, PEEP 5, *f* 20	35 ± 1°C	2		13.4 ± 1.1				23.8 ± 1.0		Humidifier inlet
				5		9.7 ± 0.6				21.4 ± 0.4		
	Dugernier, 2015 (12)	Vt 500, PEEP 5, *f* 20, Constant Inspiratory Flow 30	*N*	10	21.2 ± 1.3	16.4 ± 1.2	18.3 ± 0.2	21.2 ± 0.9			24.3 ± 1.9	15cm from ventilator
		Decelerating Inspiratory Flow 60			12.9 ± 0.4	10.7 ± 0.4	12.0 ± 0.5	16.1 ± 0.7			22.1 ± 2.0	15cm from ventilator
	Anderson, 2017 (30)	MV 100ml/kg/min, PEEP 5	37°C	4.5	7.1	3.4			17.7	19.1		Humidifier inlet
	Ge, 2019 (31)	PCV, Vt 519, MV 6.6, PEEP 4, Inspiratory flow 41	*N*	0		15.9			19.4	29.8		Humidifier inlet
		PCV, Vt 510, MV 6.9, PEEP 4		6		20.8			21.1	23.6		
		APRV, Vt 466, MV 5.3, PEEP_i_ 4		0		9.5			17.9	23.1		
		APRV, Vt 464, MV 9.5, PEEP_i_ 4		0		23.1			27.4	34.1		

*A/C*, assist/control; *PC*, pressure control; *Vt*, tidal volume (ml); *PEEP*, positive end expiratory pressure (cmH_2_O); inspiratory flow (L/min); *MV*, minute ventilation (L/min); *f*, frequency (breath/min); *Ti*, inspiratory time (s); *Te*, expiratory time (s); *N*, no; *Y*, yes; *NR*, not reported; *MV*, minute ventilation; *ETT*, endotracheal tube; *NS*, no significant.

In previous studies, i-JN was placed in completely different positions, including between the Y-piece and ETT, before the Y-piece, 15 cm from the Y-piece, and 30 cm from the Y-piece in the inspiratory limb (10, 17, 27). Only one study (17) compared two nebulizer positions and found that aerosol delivery was higher when the nebulizer was placed before the Y-piece in the inspiratory limb than between the Y-piece and the endotracheal tube (Table 1). In total, the aerosol delivery was very low, and this may be because the placement of the nebulizer and inspiratory synchronization appeared poor in the present bench study (28). In our study, the closer the nebulizer to the patient (position 1 or 2), the higher the efficiency of aerosol delivery. This can be explained by the fact that the i-JN operation, when synchronized with inspiratory airflow from the ventilator, minimizes aerosol loss during exhalation and ensures that the highest amount of medication is delivered to the patient. Niederman et al. (29) also positioned the inspiratory-synchronized VMN close to the endotracheal tube when amikacin was inhaled in mechanically ventilated patients, minimizing the area of tubing through which the aerosolized drug could be lost. However, whether adding a 15-cm extension tube has significant impact on CO_2_ rebreathing remains to be further studied.

In five previous studies on the subject with VMN, four reported that the higher nebulizer position was at the humidifier inlet/15 cm from the ventilator with bias flow during invasive ventilation (12, 13, 30, 31), and one reported that 15 cm from the Y-piece in the inspiratory limb had higher delivery without bias flow (26) (Table 1). Among the four studies with bias flow (12, 13, 30, 31), only one tested position 4 (12). We found that aerosol delivery was higher when VMN was placed in position 4 (15 cm from the Y-piece in the inspiratory limb) with a bias flow of 2 L/min. The inconsistencies between our study and a previous one (12) could be due to differences between ventilators with different bias flows and/or use (or not) of humidifier. Increasing bias flow through the ventilator circuit may decrease the amount of aerosol deposited (13). With minimum bias flow, it may be better to place the nebulizer 15 cm from the Y-piece in the inspiratory limb. In general, we may comprehensively consider bias flow, humidification, the procedure, and infection risk of adding a 15-cm tube.

To our knowledge, there is no study to compare the effects of different ventilators on aerosol delivery. We found no difference in the highest aerosol delivery among the different ventilators. However, the experiment was only conducted under one ventilator setting (PC mode) with the same bias flow, and the results need to be confirmed under more ventilator settings in further research, such as different respiratory rates, inspiratory pressures tidal volumes, and volume control modes.

Some limitations should be considered when interpreting the results of this study. First, the aerosol delivery dose was estimated *in vitro*. Many factors influence the emitted dose and make it difficult to provide objective comparisons of clinical outcomes. Additional studies with healthy volunteers or patients are warranted to establish whether the *in vitro* results are clinically relevant. We only focused on the effects of the position and nebulizer type on aerosol delivery *in vitro*. The risks and benefits of placement of nebulizers, interruption of ventilation and increase risk of VAP in patients, impact of additional mechanical dead space, impact of the position in treating patients with heat moisture exchangers (HMEs), etc., should also be considered in the clinic. We did not use an ETT to connect the ventilator circuit and the lung simulator; instead we used a 30-mm tube. Second, the behavior of the nebulizer and the distribution of particle size may be affected by the properties of the drug solution and the brand of the nebulizer tested; only one brand of jet nebulizer was used in our study. For i-JN, we did not use the nebulizer developed for the ventilator because it was not available. However, all ventilator manufacturers recommend using their own i-JN, and it is difficult to use the recommended i-JN as disposable consumables in clinical practice, but i-JN is widely used in the clinical setting. Besides, the driving flow of the c-JN we used in this study was 8 L/min, which is close to that of the two ventilators (Avea: 6 L/min, V300: 10 L/min). Third, the nebulization of JN was considered finished when there was no visible evidence of nebulization for a period of 30 s in our study, but not 1 min, which is consistent with some reports in the literature but not with international standards. Only three ventilators were used in our study with the same ventilator settings. Additional commercially available ventilators and ventilator settings need to be tested to confirm the results.

## Conclusion

During adult mechanical ventilation, positions matter in optimizing delivery efficiency. For the highest aerosol delivery in the proper nebulizer position, i-JN is marginally more efficient than c-JN, but VMN was much more efficient; If not, this phenomenon has changed. Besides, there were no differences between the ventilators with the same settings.

## Data availability statement

The original contributions presented in the study are included in the article/supplementary material, further inquiries can be directed to the corresponding author/s.

## Author contributions

HH, DX, BD, and WT conducted the study. HH, DX, and WT wrote the manuscript. DX, BD, and WT contributed to data collection. BD, HZ, WW, JK, and WT revised the manuscript and designed the study. All authors contributed to the article and approved the final version of the manuscript.

## Funding

This research was funded by the Science and Technology Planning Project, Shenyang (21-172-9-12).

## Conflict of interest

The authors declare that the research was conducted in the absence of any commercial or financial relationships that could be construed as a potential conflict of interest.

## Publisher's note

All claims expressed in this article are solely those of the authors and do not necessarily represent those of their affiliated organizations, or those of the publisher, the editors and the reviewers. Any product that may be evaluated in this article, or claim that may be made by its manufacturer, is not guaranteed or endorsed by the publisher.
